# Comparison of Selective Laser Melted Titanium and Magnesium Implants Coated with PCL

**DOI:** 10.3390/ijms160613287

**Published:** 2015-06-10

**Authors:** Julia Matena, Svea Petersen, Matthias Gieseke, Michael Teske, Martin Beyerbach, Andreas Kampmann, Hugo Murua Escobar, Nils-Claudius Gellrich, Heinz Haferkamp, Ingo Nolte

**Affiliations:** 1 Small Animal Clinic, University of Veterinary Medicine Hannover, Foundation, D-30559 Hannover, Germany; E-Mails: julia.matena@tiho-hannover.de (J.M.); hugo.murua.escobar@med.uni-rostock (H.M.E.); 2 Faculty of Engineering and Computer Science, Osnabrueck University of Applied Sciences, D-49076 Osnabrueck, Germany; E-Mail: s.petersen@hs-osnabrueck.de; 3 Institute for Biomedical Engineering, Rostock University Medical Center, D-18119 Rostock, Germany; E-Mail: m.gieseke@lzh.de; 4 Materials and Processes Department, Laser Zentrum Hannover e.V., D-30419 Hannover, Germany; E-Mail: michael.teske@uni-rostock.de; 5 Institute for Biometry, Epidemiology and Information Processing, University of Veterinary Medicine Hannover, Foundation, D-30559 Hannover, Germany; E-Mail: martin.beyerbach@tiho-hannover.de; 6 Clinic for Cranio-Maxillo-Facial Surgery, Hannover Medical School, D-30625 Hannover, Germany; E-Mails: kampmann.andreas@mh-hannover.de (A.K.); gellrich.nils-claudius@mh-hannover.de (N.-C.G.); 7 Division of Medicine Clinic III, Hematology, Oncology and Palliative Medicine, University of Rostock, D-18057 Rostock, Germany; 8 Institut fuer Werkstoffkunde, Leibniz Universitaet Hannover, D-30823 Hannover, Germany; E-Mail: haferkamp@iw.uni-hannover.de

**Keywords:** titanium implant, magnesium implant, polycaprolactone, poly-3-hydroxybutyrate, live cell imaging, osteoblast

## Abstract

Degradable implant material for bone remodeling that corresponds to the physiological stability of bone has still not been developed. Promising degradable materials with good mechanical properties are magnesium and magnesium alloys. However, excessive gas production due to corrosion can lower the biocompatibility. In the present study we used the polymer coating polycaprolactone (PCL), intended to lower the corrosion rate of magnesium. Additionally, improvement of implant geometry can increase bone remodeling. Porous structures are known to support vessel ingrowth and thus increase osseointegration. With the selective laser melting (SLM) process, defined open porous structures can be created. Recently, highly reactive magnesium has also been processed by SLM. We performed studies with a flat magnesium layer and with porous magnesium implants coated with polymers. The SLM produced magnesium was compared with the titanium alloy TiAl6V4, as titanium is already established for the SLM-process. For testing the biocompatibility, we used primary murine osteoblasts. Results showed a reduced corrosion rate and good biocompatibility of the SLM produced magnesium with PCL coating.

## 1. Introduction

Autografts are commonly used for reconstruction of critical defects in maxilla-facial orthopedics, e.g., after accidents or tumor resections [1]. The bony material can be taken from intact bone, generally out of the hip. Therefore, the patient has to undergo surgery. Furthermore, the material itself is rare and the donor site often remains painful [2,3]. To avoid additional surgery, a material with appropriate conditions comparable to bone is needed. Many different resorbable materials for bone tissue engineering have been examined for this purpose, such as poly (lactic-*co*-glycolic acid) (PLGA)-based or poly (3-hydroxybutyrate-*co*-3-hydroxyhexanoate) (PHBHHx)-based scaffolds. However, there are no commercial implants that can meet physiological forces since the early beginning of implantation and desired degradation at the same time [4,5]. Materials that potentially can fulfill these demands are magnesium and magnesium alloys since they are promising resorbable robust metals [6,7]. They are biocompatible and the elastic modulus is close to that of bone. In the last few years, studies have shown good biocompatibility of magnesium-based implants [7]. However, the gas that this material produces is a major disadvantage, lowering the bone-implant contact. Therefore, different coatings or magnesium alloys are used to lower the corrosion rate [8]. After careful consideration, we decided to use the polymer coating polycaprolactone (PCL). This synthetically produced polymer showed good results in lowering the corrosion rate of magnesium [9,10]. Both magnesium and polymer are resorbable. Magnesium provides stability, while the polymer protects the implant from initial corrosion.

Scaffold geometry can be used to enhance vascularization and consequently to achieve a good bony ingrowth. Recent studies have shown that porous scaffolds with 250 µm interconnected pores support angiogenesis [11,12]. For manufacturing the open porous structure, a selective laser melting (SLM) process was used. This rapid prototyping method offers a fast and patient individual implant production and titanium scaffolds using SLM is well established [13,14]. Recently, magnesium scaffolds have been generated using SLM as well [15]. Nevertheless, resolution of 250 µm has not been achieved yet. The manufacturing process of magnesium had to be established in this study to achieve a higher resolution and thus a smaller pore size. A flat SLM-made magnesium structure on a titanium plate coated with PCL, later referred to as magnesium hybrid construct, was manufactured and used for the first time. Furthermore, a porous magnesium implant could be manufactured with the SLM process. For comparison purposes titanium was used, because of its suitability for the SLM process and as implant material. The cell behavior on the implant materials was examined using murine green fluorescent protein (GFP) osteoblasts in the live cell imaging (LCI), as recently shown [16].

Open porous magnesium structures generated by SLM and coated with PCL could be a way to overcome the deficiency in potential biodegradable and stable material for bone implants. The herein presented work should be a first step to overcome this deficiency.

## 2. Results

### 2.1. Manufacturing

**Figure 1:**
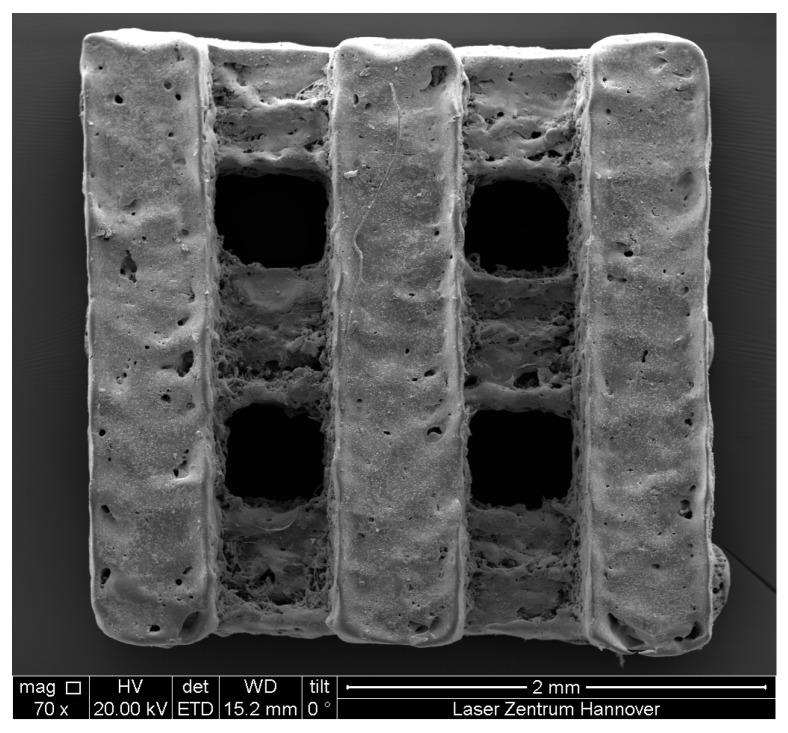
ESEM micrograph of magnesium implant manufactured by selective laser melting (SLM).

A titanium implant with 250 µm pore and strut size and a flat magnesium layer on titanium were produced by SLM and provided for *in vitro* experiments. A porous magnesium implant was manufactured of pure magnesium by SLM (Figure 1). The highest possible resolution achieved using the SLM process was 600 µm pore size.

### 2.2. In Vitro Corrosion Study

**Figure 2:**
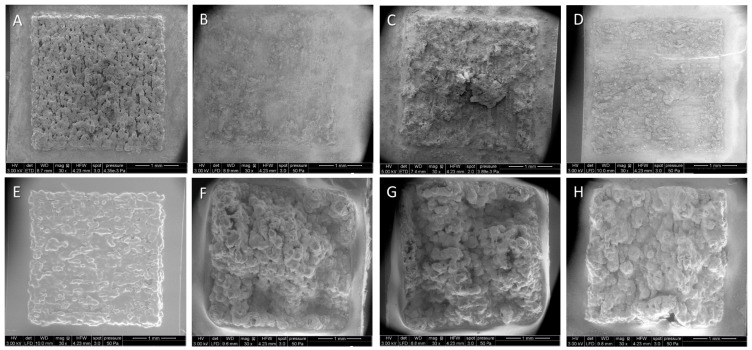
Representative ESEM micrographs of non-coated (**A–D**) and polycaprolactone (PCL)-coated (**E–H**) magnesium structures after different corrosion time intervals (**A**,**E**: 0 days; **B**,**F**: 1 day; **C**,**G**: 3 days; **D**,**H**: 21 days).

Microscopic studies were performed to evaluate the *in vitro* corrosion-induced changes in surface morphology of the non-coated and polycaprolactone (PCL)-coated flat magnesium structures processed on a titanium plate. It was observed that the non-coated magnesium structures nearly disappeared in the short corrosion time of three weeks, while the PCL-coated structures seemed even to expand (Figure 2).

**Figure 3:**
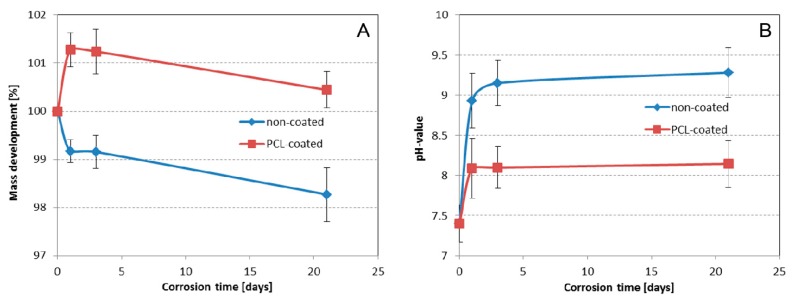
Corrosion-induced mass loss of non-coated and PCL-coated magnesium structures (**A**) and pH-value of surrounding medium (**B**) in Sørensen buffer (0.1 M, pH 7.4) at 37 °C. Data shown as means ± SD.

This observation could be confirmed by means of mass development analysis. The non-coated structures lost up to 2% of the initial total mass (SLM processed magnesium structure and titanium base structure), whereas the PCL-coated samples seemed to gain in mass (Figure 2F–H; Figure 3A).

To evaluate this result and to demonstrate the presence or absence of the PCL-coating during *in vitro* corrosion, the chemical composition of the initially PCL-coated magnesium structures was evaluated by EDX measurements. As shown in Table 1, the atomic percent (at%) of magnesium (Mg) and oxygen (O) increased, whereas the atomic percent of C decreased within the first 24 h. In the following corrosion time, no further modification of the chemical composition seemed to occur. Furthermore, the evolution of the pH values during the corrosion study was investigated. Thus, the polymer specimens were immersed in buffer without intermediate medium change. For both, non-coated and coated samples, a constant increase in the pH-value was observed, which was, however, more prominent for non-coated (highest pH: 9.3; Figure 3B) than for coated specimens (highest pH: 8.1; Figure 3B).

**Table 1:** EDX data on surface composition (atomic percent for the relevant elements Mg, O and C) of the PCL-coated magnesium structures after different corrosion intervals.

Corrosion Time (Days)	At% Mg	At% O	At% C
0	0.06	34.08	65.86
1	6.19	54.96	38.85
3	6.33	52.75	40.92
21	4.82	54.99	40.19

### 2.3. Live Cell Imaging (LCI) of Magnesium Hybrid Construct Compared with the Titanium Implant Coated with Polycaprolactone (PCL) Seeded with Green Fluorescent Protein (GFP)-Osteoblast

**Figure 4:**
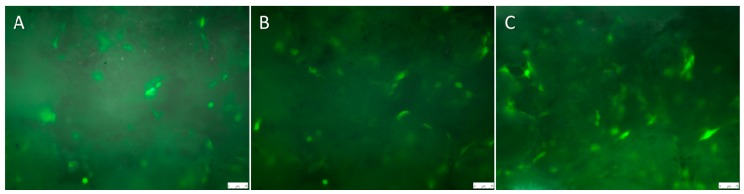
Osteoblasts seeded on the magnesium hybrid construct on Day 1 (**A**), Day 3 (**B**) and Day 7 (**C**). Scale bars: 75 µm.

Comparing cell counts after the initial seeding of osteoblasts settled on the PCL coated titanium and on the hybrid construct (Figure 4A–C), no difference could be observed.

After two days settling time, the cell count was significantly lower on the hybrid construct. We could also see a significant difference between the two scaffolds regarding the cell spreading area at the time point directly after seeding, as LCI for the PCL coated titanium implant was started after 4 h cell settling time and for the hybrid construct, after 1 h. On Day 1–7 no difference occurred in the cell spreading area. The statistical analysis was performed using the unpaired *t*-test (*p* < 0.05).

### 2.4. PCL-Thickness Measurement of Coated Magnesium Implant

**Figure 5:**
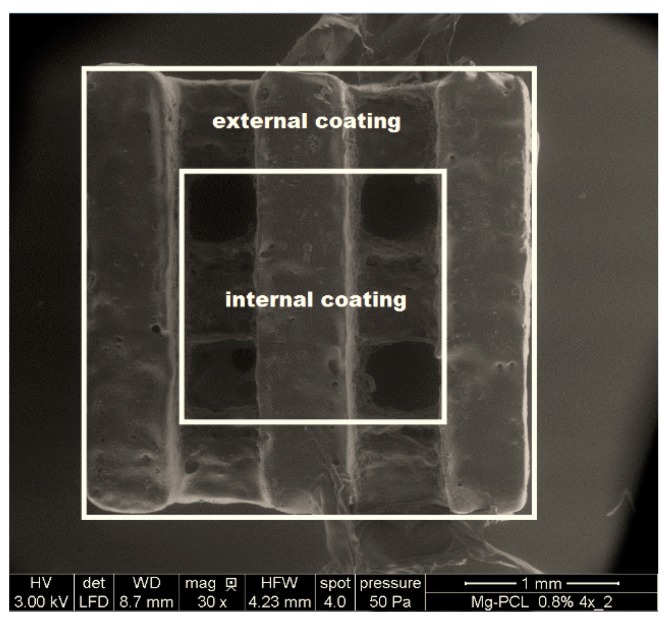
ESEM image of a magnesium PCL implant. Two different zones of the PCL coating were analyzed, named the external coating and the internal coating.

The magnesium implant was coated with PCL via a manual dip-coating process. Cross-section polishes of scaffolds were prepared to determine thickness of PCL coatings. The focus was put on the external coating, in order to evaluate the coating at the edge of the scaffold and on the internal coating with the 600 µm holes, which should enhance the angiogenesis (Figure 5). During dip coating, the direction of scaffold position was changed after each dip process in order to improve the coating homogeneity. For the external coating, a high thickness at the top, which decreases with increasing deepness of scaffold, was observed. For the internal coating a uniform thickness over the whole cross section was evaluated (Table 2).

**Table 2:** Coating thickness depending on the position of measurement.

Position	PCL External Coating (µm)	PCL Internal Coating (µm)
Top	10.8 ± 1.2	1.2 ± 0.6
Center	3.7 ± 3.5	1.3 ± 0.3
Bottom	1 ± 0.1	1.4 ± 0.3

### 2.5. LCI of Osteoblasts Seeded on Magnesium PCL and Titanium PCL Implants

Two magnesium implants sunk to the well plate bottom and thus could not be used for the further experiment. An additional video could be performed for cells seeded on titanium implants but not on magnesium implant.

**Figure 6:**
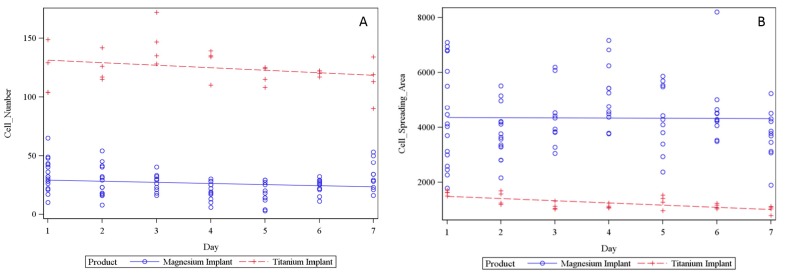
Osteoblasts seeded on titanium and magnesium implant. The cell number development (**A**) and the cell spreading area development (**B**) over seven days were examined.

Daily pictures of living cells successfully were imaged for both implant materials and were analyzed for development in cell spreading area and cell count of osteoblasts (Figure 6A,B). Comparing the results of cell spreading area and cell count, there was no difference between the two regression curves. The value of spreading area of cells settled on magnesium implant is higher than on titanium implants. Total cell numbers are higher on titanium implants in comparison with magnesium implants. A Statistical comparison test of the two regression coefficients, using an analysis of covariance, with a test of the interaction between the two implant materials and time with *p* < 0.05 was performed.

PH-changes of culture medium with magnesium implants during the experiment never extended a difference of 0.3 in comparison with medium without magnesium implant.

## 3. Discussion

The SLM process is achieved by wetting solidified material with liquid metal melted by laser radiation. Oxide layers impedes the wetting process and therefore have to be removed [17]. The titanium alloy TiAl6V4 shows a large solubility for oxygen [18], and it is assumed that oxides can be easily dissolved [19]. Thus, medical parts with an adequate surface quality can be manufactured [20]. Magnesium, in contrast, shows no solubility for oxygen [21] and also a very ductile oxide layer, which is difficult to break [22]. Using comparably high laser power, the oxide layer can be broken up and removed to facilitate the process. This results in large evaporations of magnesium, enforced by the low boiling point of magnesium. Also, regions of adhering sintered particles were found on the manufactured parts, decreasing the surface quality. Nevertheless, medical parts with an adequate surface quality can be manufactured by adjusting the scanning strategy and performing a chemical post treatment.

In general, the high corrosion rate of magnesium can be lowered by coatings [23]. PCL-coating already showed reduced corrosion for extruded magnesium rods [10]. Other studies already showed good results for bone growth using PCL [24]. In addition, PCL offers us the opportunity to incorporate growth factors, as recently shown [25]. These components are the backbone of the diamond concept suggested by Giamond *et al.* [26].

PCL coating showed promising results for corrosion resistance of magnesium and thus can be used for bone tissue engineering. As magnesium is a promising material in the field of bone reconstruction, the PCL-coated magnesium implant has great potential [7].

Therefore, a PCL coating was used with the intention of lowering the corrosion rate of the SLM-produced magnesium. A corrosion study with PCL-coated flat magnesium structures fixed on a titanium support (magnesium hybrid construct) was performed. In comparison to non-coated samples, PCL-coated samples gained in mass and increased atomic percentage of Mg and O, whereas the atomic percentage of C decreased within the first 24 h. This suggests the formation of magnesium hydroxide by water interpenetration within the polymer coating [9]; protecting complete wash off of the flat magnesium structure from the titanium support, which is the case for non-coated samples. Furthermore, the magnesium hydroxide layer seems to function as passivation of the magnesium structure since no further alteration of the surface chemical composition was observed during the following corrosion time. The more prominent increase of the pH-values for non-coated samples supports this hypothesis. Generated hydroxyl-ions during magnesium corrosion are thus kept longer on the surface of coated samples, while they are released into the surrounding media for non-coated samples resulting in greater pH. In conclusion, the biodegradable PCL-coating allows stabilization of the magnesium structure and is hence a promising candidate for alteration of the corrosion rate of magnesium-based implants.

The thickness of the PCL film on porous magnesium scaffolds seems to be inhomogeneous regarding the external coating. Some parts of the coating are very thin (under 1 µm), which have to be improved in order to ensure degradation decrease in the used scaffold structure for degradation study. For the internal coating, a uniform coating thickness for the holes were observed, only the standard deviation is marginally higher at the top of the cross-section polish. In order to improve the uniformity of these coatings, a further optimization of the magnesium scaffolds surface morphology and the coating procedures are necessary. Nevertheless, dip coating, in contrast to spray coating, seems to be suitable for porous magnesium scaffolds, as it was possible to coat the whole scaffold without closing the holes. However, for the simple geometry used for the magnesium hybrid construct, it turns out that the spray coating seems to be more suitable.

As the PCL-coating showed reduced corrosion and thus reduced pH-changes, it could be used in the cell culture. Using LCI, it was demonstrated that osteoblasts initially showed similar seeding densities for magnesium and titanium implants. After two days, cell numbers decreased on the magnesium hybrid construct. As gas production is part of the magnesium corrosion process, it is suggested that cells could be removed from the surface without effecting viability of the remaining cells; furthermore, similar cell morphology was found on both implants, the titanium implant and magnesium hybrid construct. This was confirmed by the cell spreading area, which showed a flattened cell shape from Day 1 to Day 7.

LCI of porous magnesium PCL implants obtained similar results. Nevertheless, due to the corrosion process, two of the four implants sunk to the well bottom and thus were eliminated because cells were be able to migrate towards the ground. Comparing the results of the development for cell spreading area and cell count over seven days, both magnesium PCL implant and titanium PCL implant showed equal cell behavior over time, indicated by equal slope of the regression curves. Spreading area of cells settled on magnesium implant was higher than on titanium implant. This could be due to the settings of the assay, as the fluorescence intensity was not equal due to different light reflection of the materials. Total cell number was fewer on magnesium implant. It is assumed that the corrosion process impeded cell settling, as mentioned before.

Often, magnesium is represented as supernatant to cells attached to the well plate bottom in indirect cell culture experiments [27,28]. The influence of adherence strength to study cells is missing when using indirect methods. Other studies showed growing cells in LCI settled at well plate bottoms when exposed to degraded products of magnesium [29]. Especially for magnesium, it is highly important to monitor cells directly on the implant’s surface. Due to diffusion, the chemical and physical conditions directly at the magnesium surface, in comparison to the supernatant of dissolved magnesium solution, are different. The pH on the surface could be much greater than the average pH of the solution [30].

Direct assays are performed by cell fixation and thus show one time point only [31,32,33]. The established LCI assay has the advantage of direct assays and moreover one can follow living cells over time (Video S1). Thus, we were able to compare cell settling on the magnesium hybrid construct and on the titanium implant coated with PCL. Nevertheless, no video could be recorded of the porous magnesium PCL implant, as gas production of the magnesium implant obstructed the view for cell imaging.

Open porous magnesium especially has been considered as a potential means of treating bony defects [34,35]. So far, magnesium structures having interconnected pores with 250 µm have not been made by SLM. Further investigations on SLM of magnesium and magnesium alloys showed that the use of magnesium alloys can significantly improve the SLM process.

However, the establishment of novel *in vitro* methodologies for the analyses of magnesium constructs remains key, as recent studies revealed major differences between *in vitro* and *in vivo* results limiting the predictive power of conventional *in vitro* systems for *in vivo* scenarios in general [36,37,38].

## 4. Experimental Section

### 4.1. Selective Laser Melting (SLM) of Titanium and Magnesium Implants

The titanium implants were manufactured by SLM by SLM Solutions GmbH, Luebeck, Germany from a TiAl6V4 titanium alloy.

The magnesium implants were manufactured by SLM from pure magnesium powder Atoultra 325 provided by SFM SA (Société pour la Fabrication du Magnésium), Martigny, Switzerland. An SLM125^HL^ machine system equipped with an overpressure capable process chamber provided by SLM Solutions GmbH, Germany was used to process this material. Using a laser power of 100 W, a scan speed of 3000 mm/s and an adapted hatch strategy, scaffolds with a homogenous surface quality were manufactured. Nevertheless, porous magnesium implants had to be post treated by a chemical deburring process in order to remove adhering particles and to gain smooth surfaces for sterilization and polymer coating. Micrographs of porous magnesium implant were obtained by SEM (FEI Quanta 400 FEG, FEI Company, Hillsboro, OR, USA).

### 4.2. In Vitro Corrosion Study

For investigating the *in vitro* corrosion behavior of magnesium structures prior to and after polymer coating, coated and non-coated samples (*n* = 3 samples per time point) were placed in a test tube containing 2 mL of Sørensen buffer (0.1 M, pH 7.4) and kept at 37 °C under gentle shaking. Samples were periodically removed, washed with distilled water and dried in a vacuum before analysis. The buffer solution was exchanged twice per week.

#### 4.2.1. Spray-Coating Process for Application of Polymeric Coatings to Flat Magnesium Structures for the *in Vitro* Corrosion

Flat magnesium structures were coated with PCL by means of a spray-coating process for which a specially designed spray-coating device was developed by the Institute for Implant Technology and Biomaterials (IIB e.V., Rostock, Germany). A PCL solution with concentrations of 1.53 g/L of PCL dissolved in chloroform was used. For each structure, a coating with an absolute mass of 300 µg was applied. After coating, the magnesium structures were dried in a vacuum at 40 °C for 24 h. The coating mass of the dried PCL coatings was determined by using a Mettler Toledo UMX 5 Ultra-micro Balance (Mettler-Toledo GmbH, Giessen, Germany).

#### 4.2.2. Gravimetry

To evaluate the magnesium corrosion by determining mass loss, the washed and dried samples (*n* = 3 samples per time point) were weighed using a special accuracy balance (UMX 5 Mettler Toledo, Greifensee, Switzerland). The mass loss was determined as the mass at time t divided by the initial mass multiplied by 100.

#### 4.2.3. PH Value Measurement

Polymer samples (*n* = 3) were stored in 2 mL of Sørensen buffer (0.1 M, pH 7.4) at 37 °C under gentle shaking and the pH values were measured at different time points by means of a pH meter (Seven Easy pH Meter S20, Schwerzenbach, Switzerland).

#### 4.2.4. Scanning Electron Microscopy and EDX Measurements

The surface morphology and the chemical surface composition of the PCL-coated magnesium structures were assessed after the different corrosion intervals (1, 3, and 21 days, respectively), and compared to uncoated controls using environmental scanning electron microscopy and EDX-measurements. Conditions as described above were applied.

### 4.3. Murine GFP-Osteoblast and Murine Osteoblast Isolation

Cells were isolated using adult C57Bl6 mice or GFP*C57Bl6 mice as described before [39]. Mouse calvarium was minced carefully into small pieces using 200 U/mL collagenase II (Cell Systems, Troisdorf, Germany) in Hanks’ Balanced Salt Solution (HBSS, Biochrom, Berlin, Germany) Calvarias of ten mice were pooled and 5 mL collagenase solution was added at 37 °C for 10 min and repeated five times. Only supernatant of the previous three steps was used for further centrifugation (1200 rpm, 7 min). After two washing steps with culture medium, the cells were plated and incubated at 37 °C and 8.5% CO_2_.

For further cell culture, Dulbecco’s Modified Eagle Medium (DMEM, Biochrom, Berlin, Germany) and 10% fetal calf serum (FCS), 20 mM Hepes, 1000 IU/mL penicillin and 0.1 mg/mL streptomycin (all Biochrom, Berlin, Germany) were used. Media changing was performed every third day until they were confluent.

For all further cell experiments culture medium was DMEM with 10% FCS. Conditions of incubation were 37 °C and 5% CO_2_.

### 4.4. PCL Coating of Magnesium PCL and Titanium PCL Implant

After purification of magnesium scaffolds in isopropanol, a manual dip-coating process, for which a specially designed sample holder was developed, was established. Two milliliters of polymer solutions with a concentration of 0.8% (*w*) of PCL in chloroform were used for each scaffold and filled into the dipping tanks adapted for the application. The dipping process was repeated four times with intermediate drying for 10 min at 23 ± 2 °C after each dipping process. Finally, the coated Magnesium scaffolds were dried under vacuum at 40 °C for 24 h.

The surface morphology of the PCL coated scaffolds was assessed at different sites in order to test for complete surface coverage and to compare with the non-coated control scaffolds using an environmental scanning electron microscopy (Quanta FEG 250, FEI, Eindhoven, The Netherlands) equipped with an energy-dispersive X-ray (EDX) analysis unit. The scaffolds were fixed with conductive tape on aluminum trays and the scanning electron micrographs were taken at 50 Pa pressure in a moisturized atmosphere and an accelerating high voltage of 10 kV. The presence or absence of the polymeric coatings was assessed by EDX measurements performed at the beam entrance of the electron microscope. For element (Mg, C) determination, the spectra of the fibers bombarded with electrons were analyzed.

### 4.5. PCL-Thickness Measurement of Coated Magnesium Implant

Samples were covered with a 20 nm thin gold film before embedding using Agar Sputter Coater (Plano GmbH, Wetzlar Germany). Scaffolds were embedded in epoxy resin (Epo Color™ Epoxy Resin with Epo Colour™ Epoxy Hardener, 5:1, Buehler, Waukegan, IL, USA). Cross-section polishes were prepared using a TegraPol-15 (Struers GmbH, Willich, Germany) till scratch-free. The thickness of polymer coating was determined using Microscopy (Axioskop, Carl Zeiss AG, Germany).

### 4.6. Live Cell Imaging (LCI)

#### 4.6.1. LCI of Titanium Implant Seeded with GFP-Osteoblast

Titanium scaffolds coated with PCL were placed in a 96 well plate filled with 150 µL DMEM and 10% FCS. GFP-osteoblasts of passage 9 (P 9) were added gently on the top of the scaffolds at a concentration of 2.5 × 10^4^ cells/150 μL medium in triplicate. After 5 h incubation period at 37 °C and 5% CO_2_, the implants were turned upside down to visualize the cells in the inverse microscope, and put into new wells that were prepared with purpose-built Teflon-slices used for lifting the scaffolds to create a gap between cells growing on the scaffolds and the bottom of the culture plates. Then, proliferation and motility of the cells could be observed by Live Cell Imaging Microscope (DMI6000 B, Leica Microsystems, Wetzlar, Germany) over seven days with the program LAS AF 2.6.0. For every implant, we took a picture of the same position every 15 min. A constant temperature of 37 °C was achieved by using a heating unit and CO_2_ atmosphere.

Cells at the same concentration without scaffolds and Teflon-slices were used as control. Cell count and cell size were examined by Wimasis Image Analysis GmbH.

#### 4.6.2. LCI of Magnesium Hybrid Construct and Magnesium PCL Implant Seeded with GFP-Osteoblast

After establishing LCI of titanium implants, we used magnesium implants seeded with GFP-osteoblasts. Three magnesium hybrid constructs and five magnesium PCL implants were incubated in a 6-well plate for 24 h using culture medium (DMEM with 10% FCS) and five magnesium PCL implants were incubated for 48 h. Then GFP-osteoblasts were seeded in the same concentration as before with 78,125 cells/1 cm^2^ surface area onto the scaffolds and as control on the well plate. After one hour of cell adhesion, implants were placed in the scaffold holder created for the 6-well plate. The scaffold holder was prepared to ensure a good gas exchange so that the pH value was not partially increased. Further examination was done as described above, but only for one construct each. One magnesium hybrid construct and one magnesium PCL implant was imaged at the same position for video examination. Imaging of different implants would have led to medium movement, which would have resulted in faster magnesium corrosion. Therefore, the other constructs were imaged at five different random fields every day. Cell count and cell size were examined by Wimasis Image Analysis GmbH, Munich, Germany. PH values were assessed for the surrounding medium of the magnesium implants.

### 4.7. Statistical Analysis

Statistical analyses of data were performed using SAS^®^ software, Version 9.3 (SAS Institute Inc., Cary, NC, USA). The experimental data were shown as mean ± standard deviation. The *p*-value <0.05 was considered statistically significant.

## 5. Conclusions

Magnesium implants were produced by SLM and a defined open porous structure with 600 µm was possible. PCL-coating could reduce the corrosion rate of magnesium. Nevertheless, depending on the location, the coating thickness varied and could be improved. Porous titanium implants coated with polymers as well as magnesium hybrid constructs and porous magnesium PCL implants could successfully be vitalized and examined with LCI for one week. SLM-produced magnesium, coated with PCL is a promising bone implant material and should be examined *in vivo*.

## Supplementary Materials

Supplementary materials can be found at http://www.mdpi.com/1422-0067/16/06/13287/s1.

## References

[1] Gellrich N.C., Held U., Schoen R., Pailing T., Schramm A., Bormann K.H. (2007). Alveolar zygomatic buttress: A new donor site for limited preimplant augmentation procedures. J. Oral Maxillofac. Surg..

[2] Silber J.S., Anderson D.G., Daffner S.D., Brislin B.T., Leland J.M., Hilibrand A.S., Vaccaro A.R., Albert T.J. (2003). Donor site morbidity after anterior iliac crest bone harvest for single-level anterior cervical discectomy and fusion. Spine.

[3] Sasso R.C., LeHuec J.C., Shaffrey C. (2005). Iliac crest bone graft donor site pain after anterior lumbar interbody fusion: A prospective patient satisfaction outcome assessment. J. Spinal Disord. Tech..

[4] Davies J.E., Matta R., Mendes V.C., Perri de Carvalho P.S. (2010). Development, characterization and clinical use of a biodegradable composite scaffold for bone engineering in oro-maxillo-facial surgery. Organogenesis.

[5] Yang S., Wang J., Tang L., Ao H., Tan H., Tang T., Liu C. (2014). Mesoporous bioactive glass doped-poly (3-hydroxybutyrate-*co*-3-hydroxyhexanoate) composite scaffolds with 3-dimensionally hierarchical pore networks for bone regeneration. Colloids Surf. B Biointerfaces.

[6] Willbold E., Gu X., Albert D., Kalla K., Bobe K., Brauneis M., Janning C., Nellesen J., Czayka W., Tillmann W. (2015). Effect of the addition of low rare earth elements (lanthanum, neodymium, cerium) on the biodegradation and biocompatibility of magnesium. Acta Biomater..

[7] Waizy H., Seitz J.-M., Reifenrath J., Weizbauer A., Bach F.-W., Meyer-Lindenberg A., Denkena B., Windhagen H. (2013). Biodegradable magnesium implants for orthopedic applications. J. Mater. Sci..

[8] Shadanbaz S., Walker J., Woodfield T.B., Staiger M.P., Dias G.J. (2014). Monetite and brushite coated magnesium: *In vivo* and *in vitro* models for degradation analysis. J. Mater. Sci. Mater. Med..

[9] Chen Y., Song Y., Zhang S., Li J., Zhao C., Zhang X. (2011). Interaction between a high purity magnesium surface and PCL and PLA coatings during dynamic degradation. Biomed. Mater..

[10] Xu L., Yamamoto A. (2012). Characteristics and cytocompatibility of biodegradable polymer film on magnesium by spin coating. Colloids Surf. B Biointerfaces.

[11] Ring A., Langer S., Homann H.H., Kuhnen C., Schmitz I., Steinau H.U., Drucke D. (2006). Analysis of neovascularization of PEGT/PBT-copolymer dermis substitutes in balb/c-mice. Burns.

[12] Artel A., Mehdizadeh H., Chiu Y.C., Brey E.M., Cinar A. (2011). An agent-based model for the investigation of neovascularization within porous scaffolds. Tissue Eng. Part A.

[13] Rakhmatia Y.D., Ayukawa Y., Furuhashi A., Koyano K. (2013). Current barrier membranes: Titanium mesh and other membranes for guided bone regeneration in dental applications. J. Prosthodont. Res..

[14] Wang Y., Shen Y., Wang Z., Yang J., Liu N., Huang W. (2010). Development of highly porous titanium scaffolds by selective laser melting. Mater. Lett..

[15] Jauer L., Meiners W., Poprawe R. (2013). Selective laser melting of biodegradable metals. Eur. Cells Mater..

[16] Matena J., Petersen S., Gieseke M., Kampmann A., Teske M., Beyerbach M., Escobar H.M., Haferkamp H., Gellrich N.C., Nolte I. (2015). SLM produced porous titanium implant improvements for enhanced vascularization and osteoblast seeding. Int. J. Mol. Sci..

[17] Kruth J.P., Froyen L., van Vaerenbergh J., Mercelis P., Rombouts M., Lauwers B. (2004). Selective laser melting of iron-based powder. J. Mater. Process. Technol..

[18] Murray J.L., Wriedt H.A. (1987). The O-Ti (oxygen-titanium) system. J. Phase Equilib..

[19] Louvis E., Fox P., Sutcliffe C.J. (2011). Selective laser melting of aluminium components. J. Mater. Process. Technol..

[20] Vandenbroucke B., Kruth J.P. (2007). Selective laser melting of biocompatible metals for rapid manufacturing of medical parts. Rapid Prototyp. J..

[21] Wriedt H.A. (1987). The Mg-O (magnesium-oxygen) system. Bull. Alloy Phase Diagr..

[22] Niemeyer M. (1999). Strahl-Stoff-Wechselwirkung und Resultierende Verbindungseigenschaften Beim Laserstrahlschweißen Von Magnesiumlegierungen.

[23] Zhang Y., Zhang G., Wei M. (2009). Controlling the biodegradation rate of magnesium using biomimetic apatite coating. J. Biomed. Mater. Res. Part B Appl. Biomater..

[24] Williams J.M., Adewunmi A., Schek R.M., Flanagan C.L., Krebsbach P.H., Feinberg S.E., Hollister S.J., Das S. (2005). Bone tissue engineering using polycaprolactone scaffolds fabricated via selective laser sintering. Biomaterials.

[25] Wulf K., Teske M., Lobler M., Luderer F., Schmitz K.P., Sternberg K. (2011). Surface functionalization of poly(epsilon-caprolactone) improves its biocompatibility as scaffold material for bioartificial vessel prostheses. J. Biomed. Mater. Res. Part B Appl. Biomater..

[26] Giannoudis P.V., Einhorn T.A., Marsh D. (2007). Fracture healing: The diamond concept. Injury.

[27] Hagihara K., Fujii K., Matsugaki A., Nakano T. (2013). Possibility of Mg- and Ca-based intermetallic compounds as new biodegradable implant materials. Mater. Sci. Eng. C Mater. Biol. Appl..

[28] Willbold E., Kalla K., Bartsch I., Bobe K., Brauneis M., Remennik S., Shechtman D., Nellesen J., Tillmann W., Vogt C. (2013). Biocompatibility of rapidly solidified magnesium alloy rs66 as a temporary biodegradable metal. Acta Biomater..

[29] Pichler K., Kraus T., Martinelli E., Sadoghi P., Musumeci G., Uggowitzer P.J., Weinberg A.M. (2014). Cellular reactions to biodegradable magnesium alloys on human growth plate chondrocytes and osteoblasts. Int. Orthop..

[30] Virtanen S. (2011). Biodegradable Mg and Mg alloys: Corrosion and biocompatibility. Mater. Sci. Eng. B.

[31] Wang J., Qin L., Wang K., Wang J., Yue Y., Li Y., Tang J., Li W. (2013). Cytotoxicity studies of AZ31D alloy and the effects of carbon dioxide on its biodegradation behavior *in vitro*. Mater. Sci. Eng. C Mater. Biol. Appl..

[32] Seuss F., Seuss S., Turhan M.C., Fabry B., Virtanen S. (2011). Corrosion of mg alloy AZ91D in the presence of living cells. J. Biomed. Mater. Res. Part B Appl. Biomater..

[33] Johnson I., Perchy D., Liu H. (2012). *In vitro* evaluation of the surface effects on magnesium-yttrium alloy degradation and mesenchymal stem cell adhesion. J. Biomed. Mater. Res. Part A.

[34] Witte F., Ulrich H., Rudert M., Willbold E. (2007). Biodegradable magnesium scaffolds: Part 1: Appropriate inflammatory response. J. Biomed. Mater. Res. A.

[35] Witte F., Ulrich H., Palm C., Willbold E. (2007). Biodegradable magnesium scaffolds: Part II: Peri-implant bone remodeling. J. Biomed. Mater. Res. A.

[36] Mueller W.D., Lucia Nascimento M., Lorenzo de Mele M.F. (2010). Critical discussion of the results from different corrosion studies of Mg and Mg alloys for biomaterial applications. Acta Biomater..

[37] Sanchez A.H.M., Luthringer B.J.C., Feyerabend F., Willumeit R. (2015). Mg and Mg alloys: How comparable are *in vitro* and *in vivo* corrosion rates? A review. Acta Biomater..

[38] Scheideler L., Fuger C., Schille C., Rupp F., Wendel H.P., Hort N., Reichel H.P., Geis-Gerstorfer J. (2013). Comparison of different *in vitro* tests for biocompatibility screening of Mg alloys. Acta Biomater..

[39] Chen X.D., Qian H.Y., Neff L., Satomura K., Horowitz M.C. (1999). Thy-1 antigen expression by cells in the osteoblast lineage. J. Bone Miner. Res..

